# An Impact of Prolonged Electrolysis on the Electrochemical Performance and Surface Characteristics of NiFe-Modified Graphite Electrodes for Alkaline Water Electrolysis

**DOI:** 10.3390/molecules29245820

**Published:** 2024-12-10

**Authors:** Mateusz Kuczyński, Tomasz Mikołajczyk, Bogusław Pierożyński, Mirosław Bramowicz, Sławomir Kulesza

**Affiliations:** 1 Department of Chemistry, Faculty of Agriculture and Forestry, University of Warmia and Mazury in Olsztyn, Łódzki Square 4, 10-721 Olsztyn, Poland; mateusz.kuczynski@uwm.edu.pl (M.K.); tomasz.mikolajczyk@uwm.edu.pl (T.M.); 2 Department of Materials and Machines Technology, Faculty of Technical Sciences, University of Warmia and Mazury in Olsztyn, 10-719 Olsztyn, Poland; mbramowicz@uwm.edu.pl; 3 Department of Mechatronics, Faculty of Technical Sciences, University of Warmia and Mazury in Olsztyn, 10-719 Olsztyn, Poland; slawomir.kulesza@uwm.edu.pl

**Keywords:** NiFe-modified electrodes, alkaline water electrolysis, catalyst stability, surface characterization, SEM/EDS, XRD

## Abstract

This study investigates the influence of prolonged electrolysis on the electrochemical performance and surface characteristics of NiFe-modified compressed graphite electrodes used in alkaline water electrolysis. The electrochemical experiment was conducted over a two-week period at a constant temperature of 60 °C. The electrodes were evaluated for changes in surface morphology and composition using scanning electron microscopy (SEM), energy-dispersive X-ray spectroscopy (EDS), and X-ray diffraction (XRD). The results demonstrated stable electrochemical performance with minimal current variation. However, significant structural changes occurred, including the formation of new microstructures on the cathode and the emergence of KHCO_3_ (potassium bicarbonate) compound on both electrodes. Crystallographic analysis revealed an increase in crystallite size and tensile lattice strain on the cathode, while the anode exhibited compressive lattice strains and a reduction in crystallite size. These findings suggest that the observed changes were driven by electrochemical annealing processes, contributing to material redistribution and surface modifications during prolonged electrolysis. This study provides insight into optimizing NiFe-based catalysts for enhanced durability and efficiency in water splitting technologies.

## 1. Introduction

The global transition to sustainable energy has intensified interest in hydrogen production via water splitting, particularly through alkaline water electrolysis (AWE). This process is favored for its cost-effectiveness and reliance on non-precious metal-based catalysts. A critical challenge in AWE is enhancing the efficiency of the oxygen evolution reaction (OER), which requires stable and efficient catalysts. NiFe-based catalysts have emerged as promising solutions due to their high activity, low overpotential (~200 mV), and use of earth-abundant materials. Over time, these catalysts have evolved from simple bimetallic systems to advanced materials, such as layered double hydroxides (LDHs) and transition metal phosphides (TMPs) and sulfides (TMSs). These materials leverage the synergistic interaction between Ni and Fe to improve electronic conductivity and accelerate the OER kinetics. The synergistic effect arises as nickel provides an active site for oxygen evolution, while iron enhances the electronic structure and modulates the oxidation state of nickel, leading to improved catalytic efficiency [1,2,3,4,5,6,7,8,9,10,11,12,13,14].

LDH-based NiFe catalysts are particularly attractive due to their highly tunable layered structure, which offers an abundance of exposed active sites and excellent ion transport properties. These features contribute to their superior OER kinetics in alkaline media. Additionally, recent advancements have focused on incorporating dopants, such as Co or Mn, into the LDH structure to further enhance its catalytic activity and stability [2,13,15].

On the other hand, TMPs and TMSs are valued for their intrinsic high electrical conductivity, which facilitates charge transfer during the oxygen evolution reaction. Under OER conditions, these catalysts undergo in situ transformations into oxyhydroxide phases, such as NiOOH and FeOOH, which act as the true active species. The presence of dopants such as phosphorus or sulfur in TMPs and TMSs enhances this transformation by adjusting the valence states of Ni and Fe, leading to optimal electronic configurations for enhanced catalysis. These dopants also reduce the energy barriers for key OER steps, thereby increasing the overall reaction rate [16,17].

Moreover, the self-healing nature of the NiFe catalysts, particularly the dissolution-redeposition dynamics of iron, has been highlighted as a crucial factor in maintaining catalytic performance during prolonged operation. The formation of [FeO_4_]^2−^ complexes in alkaline media, followed by their redeposition on the catalyst surface, stabilizes the active sites and prevents material loss. This dynamic equilibrium is essential for improving the durability of the NiFe catalysts under real-world operating conditions. However, their long-term stability under extended electrolysis conditions still remains insufficiently understood [12].

LDH NiFe catalysts are particularly notable for their rapid OER kinetics and adjustable surface properties, while TMPs and TMSs offer excellent conductivity and enhanced performance through sulfur or phosphorus doping. Under the OER conditions, these materials undergo in situ surface transformations, forming active oxyhydroxide phases which can maintain or even improve catalytic activity. These dynamic transformations, driven by dissolution and redeposition processes, create a self-healing effect which stabilizes the catalyst’s surface [13,14,18,19].

While these initial transformations have been extensively studied [13,14,18,19,20,21,22], the long-term effects of prolonged electrolysis on the NiFe catalysts remain underexplored [23,24]. Changes in morphology, chemical composition, and structural integrity over extended electrolysis could significantly influence both the durability and electrochemical efficiency of the electrodes. Although these long-term changes are critical for practical applications, they have received comparatively less attention. Understanding the structural and compositional evolution of the NiFe-modified electrodes during extended electrolysis is crucial for improving the catalyst’s longevity and optimizing the overall performance of water splitting systems.

This study aims to evaluate the long-term effects of alkaline water electrolysis on the surface characteristics and electrochemical performance of NiFe-modified electrodes from our recent publication [25]. This investigation provides new insights into optimizing electrode materials for extended operation in hydrogen production technologies.

## 2. Results and Discussion

### 2.1. Electrochemical Characteristics

**Figure 1:**
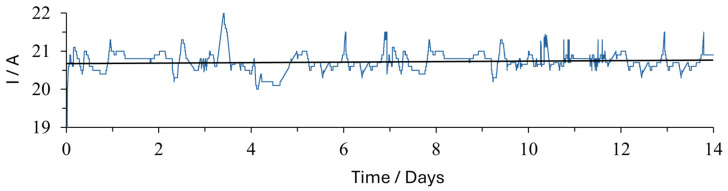
Electrolyzer stack’s current as a function of the electrolysis time. The process was carried out at 60 °C in 8 M KOH solution for the set voltage value of 10 V.

The continuous alkaline water electrolysis experiment demonstrated stable electrochemical performance over a two-week period. Initially, for the set voltage of 10.0 V (about 2.5 V per single cell), the current rose steadily from about 6.0 to 20.0 A within the first 30 min of the stack operation. After an initial rise, the current fluctuated between roughly 20.0 and 22.0 A, with a mean value of 20.73 A. A linear trend (with a minimal increase in current) was observed over prolonged electrolysis (Figure 1). This suggests that while there was some current variation, the system maintained steady operation throughout the duration of the experiment. This consistent performance highlights the stability of the system, particularly the use of compressed graphite electrodes with NiFe catalyst loading at 0.5 mg cm^−2^, which effectively sustained electrochemical activity under prolonged operating conditions at 60 °C. Compared with the results from a previous study [17] using Ni foam electrodes, which achieved a current of about 21.0 A and a voltage of 2.65 V per cell, the compressed graphite-based electrodes exhibited slightly better performance in terms of the lower operating voltage [26]. Moreover, although the Pd-modified Ni foam electrodes reported in the same study demonstrated a superior current of 23.8 A at 2.65 V per cell, the compressed graphite electrodes exhibited better stability. The voltage for the Pd-modified Ni foam stack system increased from 21.0 to 23.8 V after 7 days, indicating a reduction in efficiency over time, while in this work, the compressed graphite maintained steady performance throughout the entire experiment.

In contrast to compressed graphite, expanded graphite, which was also used in our recent publication [25], exhibited significant instability here during the early stages of the prolonged electrolysis. The latter primarily resulted from the fact that the current density (roughly 200 mA cm^−2^) imposed in this work was about an order in magnitude higher than that employed in [13]. Moreover, expanded graphite material, due to its structure, is much more susceptible to electrolyte penetration (and thus accelerated disintegration) than its compressed graphite analogue. Thus, the authors of this article were prompted to exclusively focus these long-time AWE studies on the employment of compressed graphite base electrode material.

### 2.2. Structure Analysis

**Figure 2:**
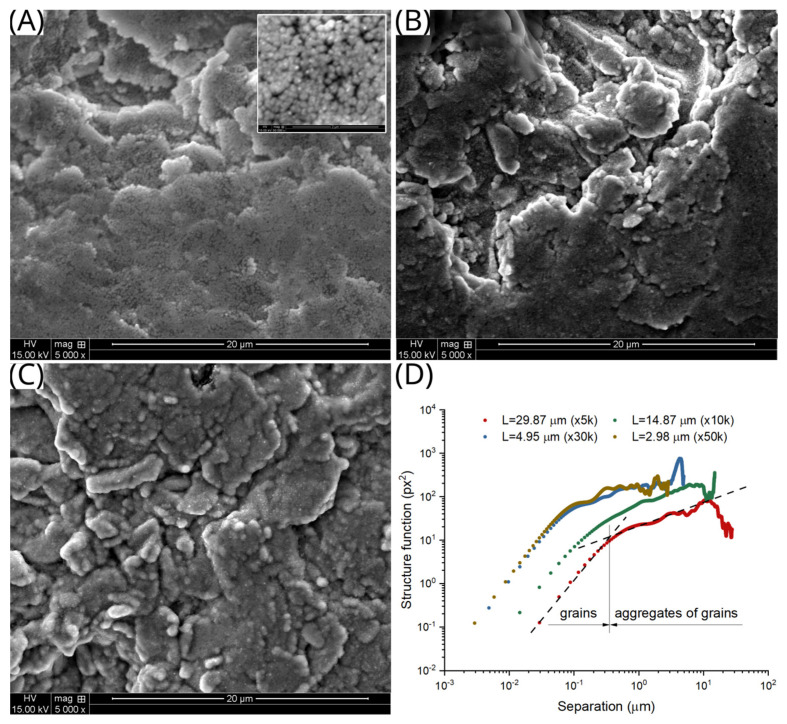
SEM images of the surfaces of the electrode materials under 5 k magnification: (**A**) NiFe-modified graphite (inset: the same surface under 50 k magnification, revealing the granular structure of the graphite flakes), (**B**) cathode, (**C**) anode, and (**D**) examples of log-log plots of structure functions derived for graphite with NiFe from SEM images under various magnifications. On the lowest magnification image (×5 k), straight lines are shown corresponding to different fractal dimensions D1 and D2, separated by kinks which establish the corner frequencies.

Following the continuous two-week electrolysis experiment, the surface morphologies of both the anode and cathode materials were analyzed by means of scanning electron microscopy (SEM) supplemented by an energy-dispersive X-ray spectroscopy (EDS) device. The SEM images (Figure 2A–C and Table 1) revealed the polycrystalline morphology of the samples.

**Figure 3:**
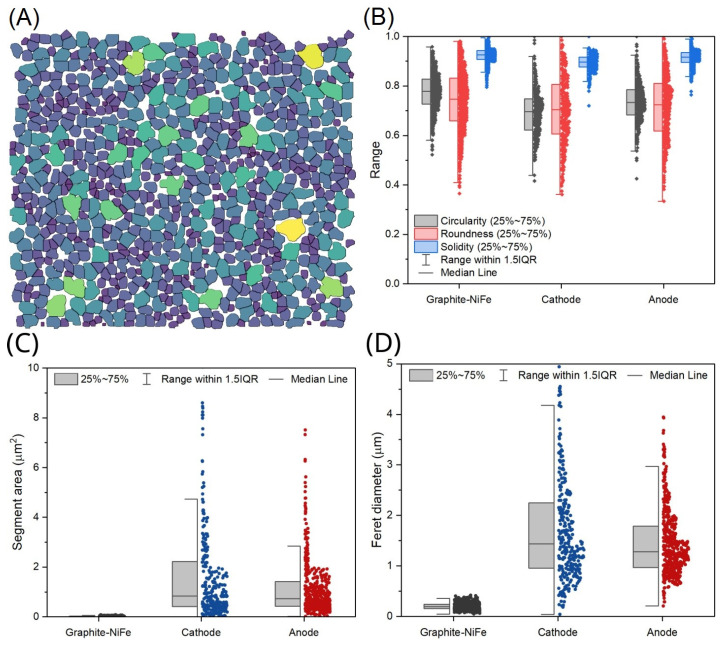
Results of stereometric characterization of SEM images: (**A**) segmented SEM image of graphite with NiFe from Figure 2A’s inset, (**B**) comparison of stereometric shape qualifiers (circularity, roundness, and solidity), (**C**) comparison of the areas of the segments, and (**D**) comparison of Feret’s diameter for the segments.

**Figure 4:**
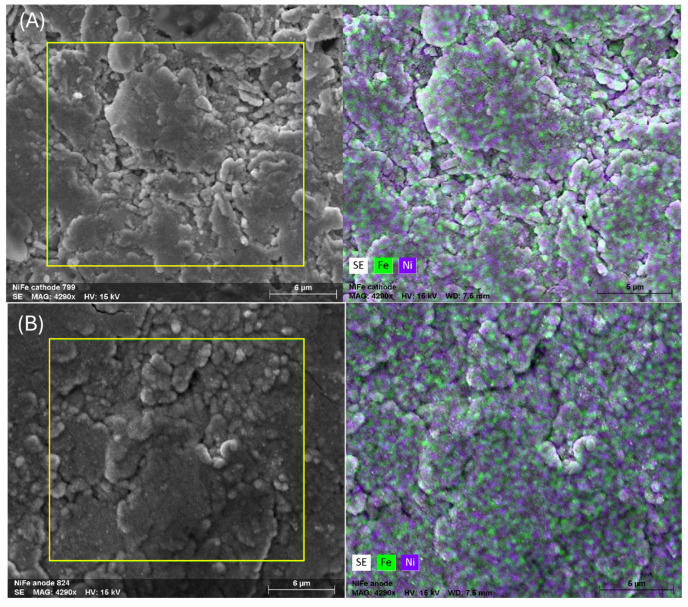

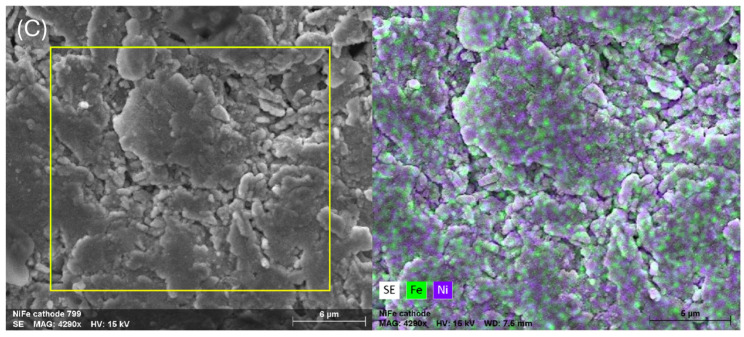
SEM micrograph pictures of NiFe-modified compressed graphite before (**A**) and after electrolysis for the (**B**) anode and (**C**) cathode, taken at 5000× magnification with EDX elemental mappings.

The fractal analysis performed on the SEM images (as an example, see Figure 2D, showing a series of log-log plots of the structure functions obtained under various magnifications from the same sample) demonstrated that both the anode and cathode deposits exhibited bifractal behavior, which is typical of surfaces composed of regular grains forming larger clusters. The size of the individual grains and clusters varied significantly between the cathode and anode. Prior to electrolysis, the grains on the NiFe-modified graphite measured approximately 240 nm in diameter, with clusters reaching sizes of about 4600 nm. After two weeks of continuous electrolysis, substantial growth was observed. On the cathode, grains expanded to roughly 300 nm, and the clusters reached nearly 9300 nm in size. Meanwhile, the anode surface exhibited grains measuring up to 400 nm, with clusters expanding to approximately 7000 nm. This growth of grains across the electrodes suggests a complex process of material redistribution, possibly due to the so-called electrochemical annealing process [27,28], rather than recrystallization induced by thermal effects given the relatively low temperature of 60 °C during the process. Electrochemical annealing occurs when the applied potential promotes atomic mobility on the electrode surface, leading to structural rearrangements and surface smoothing similar to thermal annealing but driven by electrochemical factors. This process allows defects to “heal” and atoms to reorganize into more stable configurations, resulting in the observed material redistribution. Interestingly, the observed structural changes (e.g., see Figure 3B–D) did not adversely affect the catalytic performance of the examined electrodes. Specifically, the electrochemical annealing provided stabilization of the catalyst’s surface along with preservation of a uniform distribution of the NiFe catalyst, which was confirmed by EDS and SEM analyses (see Figure 4A–C).

Note that the prime fractal dimension D_1_ slowly decreased from sample to sample, being accompanied by increasing corner frequency τ_1_, which might be interpreted as a subtle flattening of the surface of the tiniest grains when they grow up. On the other hand, there was no similar correlation in terms of the clusters of the grains when considering the higher-order fractal dimension D_2_ and corresponding corner frequency τ_2_.

Additionally, stereometric analysis was applied to quantify shape descriptors such as circularity, roundness, solidity, and Feret’s diameter, which are presented in Figure 3 and Table 2. The geometric characteristics, such as circularity (ranging from 0.691 to 0.776), roundness (0.703–0.739), and solidity (0.894–0.923) did not significantly change between the samples, suggesting that the grain shapes remained stable throughout the electrolysis process. Thus, the grains retained regular, slightly oval-like shapes with smooth edges.

However, notable differences were observed in the grain sizes. Initially, small grains with a diameter of approximately 200 nm and an area of about 20 µm^2^ were detected. Then, during the electrolysis, the grains exhibited somewhat similar growth, with grains 1.8 µm in diameter and an area of 1.9 µm^2^ forming on the cathode compared with the anode ones, having a 1.5 µm diameter and an area of 1.2 µm^2^. The significant growth of these grains was also likely influenced by the electrochemical annealing taking place during the process of electrolysis. Additionally, the grain size distribution was wide, indicating significant variability across the samples.

The EDX analysis (Figure 4 and Table 3) provided further insights into the elemental composition of the electrodes, confirming the presence of a NiFe entity on the surface of the electrodes. Moreover, the surface deposition of NiFe was observed to be quite regular, indicating a uniform distribution of the catalyst material across the surface.

Additionally, the analysis revealed a similar shift in the ratio of nickel to iron on both the anode and cathode surfaces compared with the NiFe-modified electrode before the electrolysis process. Specifically, the amount of Ni decreased, while the amount of Fe increased. This outcome is somewhat unexpected, as a typical dissolution of the anode followed by the deposition of dissolved cations on the cathode would likely result in different Ni:Fe ratios between the electrodes. The latter implies that a different mechanism may be influencing this change, possibly the formation of new structures during the electrolysis process, which affected the EDS readings. Further clarification of this observation will be provided in the X-ray diffraction (XRD) analysis section.

**Figure 5:**
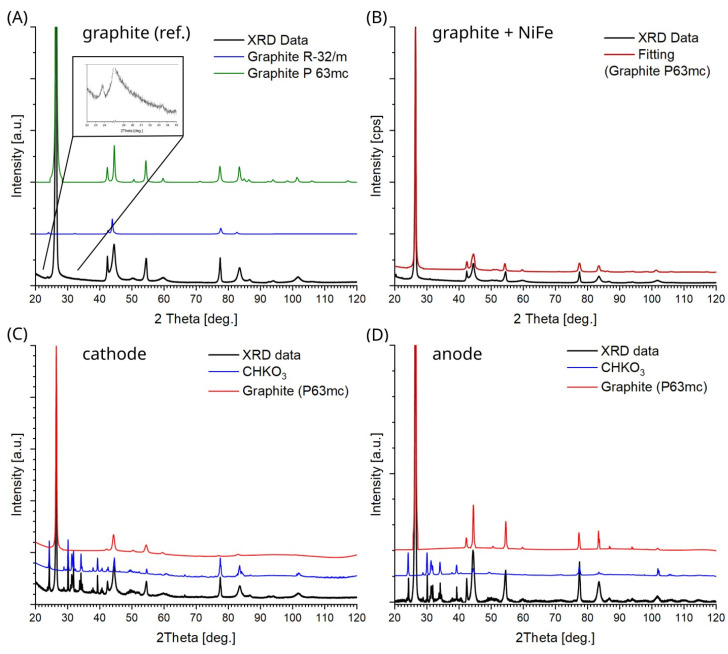
XRD spectra of the electrode materials (**A**) for unmodified Graphite; (**B**) for NiFe-modified graphite before electrolysis; (**C**) for cathode; (**D**) anode.

In order to further elucidate the structural changes induced by the prolonged alkaline water electrolysis, X-ray diffraction (XRD) analysis was performed on the electrode materials. The XRD spectra (Figure 5) of the reference sample (unmodified graphite) revealed the predominant presence of the hexagonal graphite phase with P63mc symmetry (space group 186). Additionally, a minor phase corresponding to trigonal graphite with R-3m symmetry (space group 166) was detected, comprising approximately 4% by volume (Table 4). This trigonal phase exhibited characteristic peaks at 23.951° for the (009) plane and 33.121° for the (00.12) plane (Table 5).

After the NiFe electrodeposition, the recorded XRD patterns indicated a notable structural transformation (Table S1 in the Supplementary Materials). The trigonal graphite phase, evident in the pure graphite sample, was no longer observed, as marked by the disappearance of the (009) peak at 23.951°. This suggests that the incorporation of Ni and Fe atoms disrupted the long-range order of the trigonal graphite phase. The authors suggest that rather than forming distinct intermetallic phases, which were below the detection threshold due to their low volume content, the Ni and Fe atoms may have diffused into the graphite lattice to form interstitial solutions.

Furthermore, the XRD analysis revealed the presence of a small amount (approximately 1% by volume) of KHCO₃, which appeared after the electrolysis. This compound was detected on the electrode surfaces, as indicated by additional peaks in the XRD spectra. The formation of KHCO₃ (potassium bicarbonate) was directly related to the chemical reaction of KOH, with CO_2_ molecules migrating into the electrolyte upon electrolysis.

Crystallographic analysis using the Williamson–Hall method revealed significant differences between the cathode and anode materials. For the cathode, the crystallite size increased by a factor of four, accompanied by a sixfold increase in tensile lattice strain. Conversely, the anode material exhibited compressive lattice strains of approximately 0.2% and a 30.0% reduction in the crystallite size, indicating distinct mechanical responses to electrolysis at the anode and cathode electrodes.

## 3. Materials and Methods

### 3.1. Electrode Stack

**Scheme 1:**
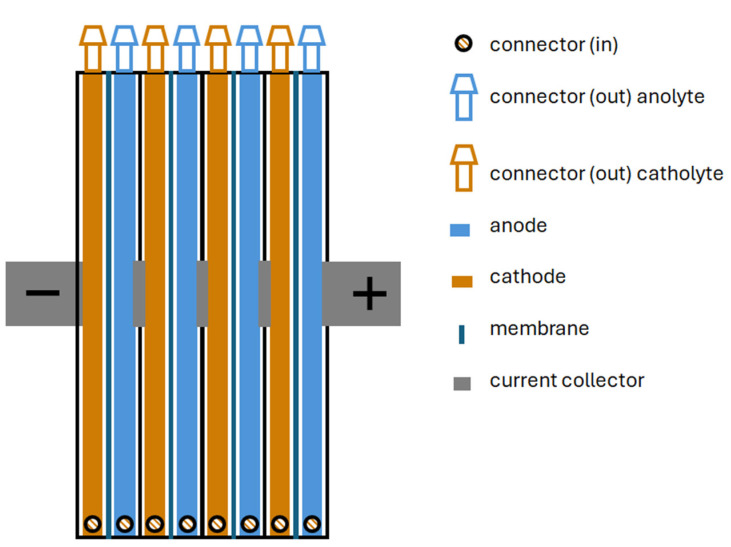
Electrolyzer’s electrode stack.

**Scheme 2:**
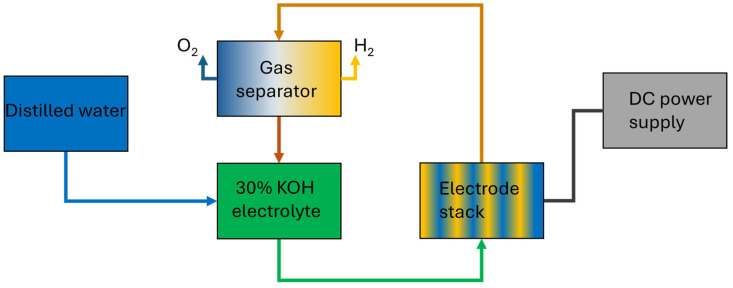
Alkaline water electrolyzer system.

The electrochemical performance of the electrodes was evaluated by means of a laboratory-made electrolyzer stack consisting of four cells connected in series, with a total applied voltage of approximately 10.0 V (about 2.5 V per cell) (with DC-power supply PeakTech, Ahrensburg, Germany). The electrolyzer stack’s design and system construction are detailed in Scheme 1 and Scheme 2. A constant temperature of 60 °C was maintained during the two-week electrolysis, and 30 wt.% KOH electrolyte (prepared form 99.9% pure granulate, Sigma-Aldrich, Poznań, Poland) was recirculated using a peristaltic micro-pump (Aqua-Trend, Łódź, Poland) at a flow rate of 600 mL min^−1^. The compressed graphite (IEG-45, Sinograf, Toruń, Poland) electrodes (10 × 10 cm) with NiFe catalyst loading of 0.5 mg cm^−2^ were exclusively used, following the preparation procedure depicted in detail in our previous study [25]. Hydrogen and oxygen gases produced during electrolysis were continuously eliminated from the solution using a gas separator module in order to ensure their efficient removal. The distilled water supply for the electrolyzer unit was automated, delivering 20 mL hour^−1^ of H_2_O with slight manual adjustments, executed twice a day to ensure consistent electrolyte levels. The electrochemical performance was monitored by measuring the current intensity every minute throughout the entire experiment.

After completing the two-week electrolysis process, the electrodes were subjected to a comprehensive analysis to evaluate the influence of prolonged electrolysis on their morphology, commencing with a macro-scale assessment and progressing to nanoscale investigation.

### 3.2. Structure Analysis Methods

#### 3.2.1. Scanning Electron Microscopy/Energy-Dispersive X-Ray Spectroscopy (SEM/EDS)

The surface morphology and elemental composition of the NiFe-modified compressed graphite electrodes were examined at the microscale using a Quanta FEG 250 (FEI Company, Brno, Czech Republic) microscope equipped with Bruker XFlash 6010 EDX instrumentation (Berlin, Germany), offering a resolution of 125 eV. These analyses were carried out to assess changes in the surface features and elemental distribution after prolonged electrolysis, providing insights into the structural integrity of the NiFe catalyst.

The polycrystalline morphology of the electrode surfaces was examined using stereometric analysis. To identify individual segments, SEM images were binarized using the watershed algorithm. The shape descriptors calculated included circularity, roundness, solidity, and Feret’s diameter [29]. Circularity measures how close a shape is to a perfect circle, while roundness refers to the ratio of the minor axes to the major axes of the best fit ellipse. Solidity, the ratio of the area to its convex hull, indicates convexity, and the Feret’s diameter is the longest distance between any two points on a segment’s boundary. These stereometric characteristics were compared with the results from the structure function and fractal analysis to provide a multiscale surface characterization, which has been shown to offer advantages over traditional statistical measures [30,31]. Grayscale SEM images were used to derive mean profiles *z*(*x*)** through line-wise averaging. These profiles were then used to calculate the structure function, defined as

(1) $$S_{k}=\frac{1}{N-k}\sum_{i=1}^{N-k}{\left(z_{i+k}-z_{i}\right)}^{2}$$

where *z_i_* is the *i*th sample in the mean profile and *r* is the separation distance (or lag) between the samples.

The structure function of the continuous separation variable *r* can be interpreted in terms of the scale-dependent roughness parameters and correlation lengths:

(2) $$S\left(r\right)=2S_{q}^{2}\left[1-\exp(-{\left(r/\xi \right)}^{2\alpha })\right]$$

where *S_q_*^2^ is the long-range root mean square (rms) surface roughness, *α* is the Hurst exponent, and *ξ* is the horizontal correlation length. Note that for *r* >> *ξ*, the structure function reaches a saturation value of 2*S_q_*^2^, corresponding to the uncorrelated surface roughness. On the other hand, in the limit *r* << *ξ*, the structure function is reduced to first-order terms of its Taylor expansion, leading to the following allometric relation:

(3) $$S\left(r\right)=K\cdot r^{2\alpha }=K\cdot r^{2\left(2-D\right)}$$

where *D* is the fractal dimension and *K* is a scaling factor known as topothesy. On a log-log plot, this allometric dependence produces a constant slope, indicating scale invariance. Sharp variations in the slope reflect higher-order topography patterns, due to aggregated structures on the multifractal surfaces.

#### 3.2.2. X-Ray Diffraction (XRD) Analysis

The crystalline structure of the electrode materials was investigated by means of the X-ray diffraction (XRD) method to assess changes induced by the electrolysis process. The XRD measurements were performed using a Phaser D2 instrument (Bruker, Karlsruhe, Germany) in the 2θ range from 20° to 120°, with a step increment of 0.01°. The X-ray beam was generated using a copper tube, providing Kα radiation with an averaged wavelength of λ CuKα = 1.54184 Å.

Several analysis methods were applied to extract structural information from the XRD data, which are described below.

#### 3.2.3. Rietveld Refinement Method

**Figure 6:**
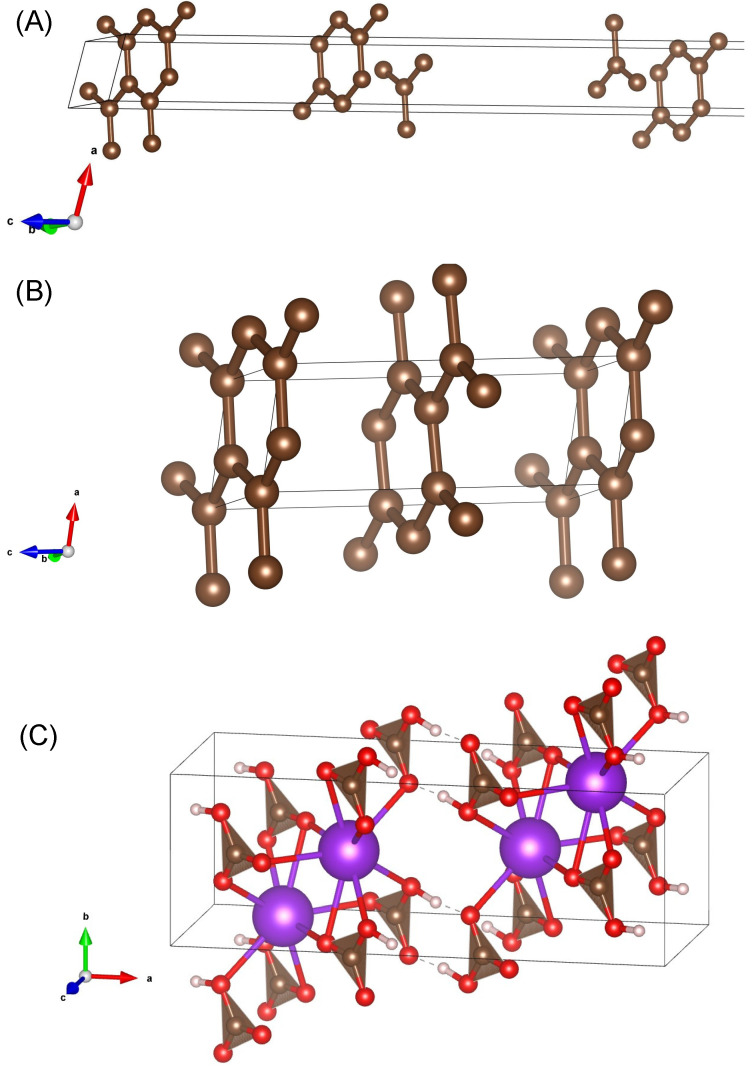
Structural models of unit cells for (**A**) graphite SG186; (**B**) graphite SG166; and (**C**) KHCO_3_, with visible K^+^ cation coordination to eight oxygen atoms (e.g., see [24] for further details).

The Rietveld method was used to quantify the crystalline phases present in the electrode materials. This method assumes that the XRD spectrum is a superposition of the spectra of individual phases, with their contributions proportional to the volume content. By using self-consistent nonlinear fitting, the lattice constants and relative volume contents of the crystalline phases were refined [32]. The starting parameters used in the Rietveld analysis, along with the corresponding structural models, are presented in Table 5 and illustrated in Figure 6.

#### 3.2.4. Scherrer Method

The Scherrer method was employed to estimate the size of the crystallites from the broadening of the diffraction peaks. The size of the diffracting domains (crystallites) was calculated using the following equation [33]:

(4) $$D_{hkl}=\frac{K\cdot \lambda }{\beta \cdot \cos\left(\theta_{hkl}\right)}$$

where *θ_hkl_* is the glancing angle, *β* is the FWHM of the peak, *K* is the shape factor (usually 0.89), and *λ* is the averaged wavelength of the X-ray beam.

#### 3.2.5. Williamson–Hall Method

The Williamson–Hall method was used to distinguish between peak broadening caused by the crystallite size and the lattice strain. This method extends the Scherrer equation by considering the contribution of the lattice strain (*ε*) to the peak broadening [34]:

(5) $$\beta \cdot \cos\left(\theta_{hkl}\right)=\frac{K\cdot \lambda }{D_{hkl}}+4\cdot \varepsilon \cdot \sin\left(\theta_{hkl}\right)$$

## 4. Conclusions

The results of this study demonstrate that prolonged alkaline water electrolysis induces significant structural changes in NiFe-modified electrodes, contributing to both material redistribution and surface modifications. The electrochemical performance remained stable over the two-week period, with minimal fluctuations in the recorded current, indicating that the NiFe-modified compressed graphite electrodes could sustain stable operation under the prolonged electrolysis conditions.

The carried out post-electrolysis surface analyses revealed an increase in surface roughness on the cathode, accompanied by the formation of new microstructures. The emergence of a KHCO₃ entity on the electrode surfaces during the electrolysis is related to the process of continuous synthesis of potassium hydrogen carbonate species, which should actually be minimized in order to avoid possible detrimental effects related to electrode or membrane contamination. The crystallographic analysis showed distinct differences between the anode and cathode materials, where the cathode exhibited a fourfold increase in crystallite size and significant tensile lattice strain, while the anode demonstrated compressive strain and a reduction in the crystallite size. These changes were likely driven by the electrochemical annealing effect, which caused significant atomic mobility and rearrangement on the electrode surfaces.

These findings suggest that prolonged electrolysis leads to important structural transformations which could positively impact the catalyst’s HER (or OER) performance.

## Figures and Tables

**Table 1:** Fractal parameters derived from SEM images of the electrode materials, where D_1_ and D_2_ are fractal dimensions and τ_1_ and τ_2_ are the corner frequencies.

Sample	D_1_ (-)	D_2_ (-)	τ_1_ (µm)	τ_2_ (µm)
Graphite/NiFe	1.068	1.668	0.061	0.797
Cathode	1.042	1.618	0.299	9.273
Anode	1.023	1.770	0.386	6.972

**Table 2:** Averaged stereometric parameters obtained through segmentation of SEM images.

Sample	Area (µm^2^)	Circularity (-)	Roundness (-)	Solidity (-)	Feret Diameter (µm)
Graphite/NiFe	0.021 ± 0.013	0.776 ± 0.073	0.739 ± 0.121	0.923 ± 0.029	0.200 ± 0.062
Cathode	1.900 ± 2.817	0.691 ± 0.093	0.703 ± 0.137	0.894 ± 0.034	1.764 ± 1.173
Anode	1.189 ± 1.497	0.734 ± 0.078	0.718 ± 0.127	0.913 ± 0.031	1.459 ± 0.722

**Table 3:** Averaged elemental composition.

	Element	C (wt. %)	Ni (wt. %)	Fe (wt. %)	O (wt. %)	K (wt. %)	Sum (wt. %)
Sample							
Graphite/NiFe		6.87	74.01	7.72	11.41	-	100.00
Cathode		7.30	67.59	12.70	9.17	3.25	100.00
Anode		5.19	67.77	15.64	9.17	2.24	100.00

**Table 4:** Lattice constants and volume contributions of the crystalline phase components seen in the electrode materials, estimated using the Rietveld method.

Sample	Crystalline Phase								
	Graphite (P63mc, Sg.186)				Graphite (R-3m, Sg.166)				
	a_0_	c_0_	c_0_/a_0_	Vol (%)	a_0_	c_0_	c_0_/a_0_	Vol (%)	
Graphite	2.4671	6.7579	2.7392	96	2.4601	33.4118	13.6	4	
Graphite/NiFe	2.4677	6.7621	2.7402	100	-	-	-	-	
	**Graphite** **(P63mc, Sg.186)**				**KHCO_3_**				
	**a_0_**	**c_0_**	**c_0_/a_0_**	**Vol (%)**	**a_0_**	**b_0_**	**c_0_**	**β_0_**	**Vol (%)**
Anode	2.4745	6.7473	2.7267	98	15.2209	5.6236	3.7051	104.8527	2
Cathode	2.4800	6.7465	2.7204	99	15.1743	5.6286	3.7133	104.6331	1

**Table 5:** Summary of structural parameters used in the Rietveld analysis.

Phase	Symmetry, Starting Lattice Constant	Atom	Coordinates of Atoms			Cell Volume (Å^3^)	Atoms per Unit Cell
			x	y	z		
Graphite	P63mc (No 186), a_0_ = b_0_ = 2.47 Å c_0_ = 6.79 Å	C	0 0.3333	0 0.6667	0 0.0050	35.875	4
Graphite	R-3m (No 166), a_0_ = b_0_ = 2.47 Å c_0_ = 6.79 Å	C	0 0	0 0	0.5 0.283	175.307	12
KHCO_3_	P21/c (No 14), a_0_ = 151,725 Å b_0_ = 56,283 Å c_0_ = 37,110 Å β_0_ = 104,631^o^	C	0.11967	0.515	−0.14363	306.627	24
		H	0.011	0.682	−0.465		
		K	0.16533	0.02177	0.29533		
		O	0.19329 0.08221 0.07761	0.52915 0.31998 0.71772	0.09482 −0.27353 −0.27448		

## Data Availability

Dataset available on request from the authors.

## Supplementary Materials

The following supporting information can be downloaded at https://www.mdpi.com/article/10.3390/molecules29245820/s1. Table S1: Various lattice parameters of the electrode materials obtained by means of the Scherrer and Wil-liamson-Hall methods, where: (hkl)—is Miller index, 2θ—is Braggs’ angle, FWHM—is full width at half maximum of the peak, DS—is size of the crystallites evaluated using Scherrer method, DWH—is size of the crystallites using Williamson-Hall method, ε—is relative lattice strains.
