# Supplementary material for: An Impact of Prolonged Electrolysis on the Electrochemical Performance and Surface Characteristics of NiFe-Modified Graphite Electrodes for Alkaline Water Electrolysis
Source: Molecules. 2024 Dec 10;29(24):5820. doi: 10.3390/molecules29245820 (PMC11676974; doi:10.3390/molecules29245820)
Supplement: Supplementary file 1 [file molecules-29-05820-s001.zip › molecules-3325235-supplementary.pdf]

Supplementary file

An Impact of Prolonged Electrolysis on the Electrochemical Performance and Surface Characteristics of NiFe-Modified Graphite Electrodes for Alkaline Water Electrolysis

Mateusz Kuczyński [1], Tomasz Mikołajczyk [1], Bogusław Pierożyński [1,*], Mirosław Bramowicz [2] and Sławomir Kulesza [3]

[1] Department of Chemistry, Faculty of Agriculture and Forestry, University of Warmia and Mazury in Olsztyn, Łódzki Square 4, 10-721 Olsztyn, Poland; mateusz.kuczynski@uwm.edu.pl (M.K.); tomasz.mikolajczyk@uwm.edu.pl (T.M.)

[2] Department of Materials and Machines Technology, Faculty of Technical Sciences, University of Warmia and Mazury in Olsztyn, 10-719 Olsztyn, Poland; mbramowicz@uwm.edu.pl

[3] Department of Mechatronics, Faculty of Technical Sciences, University of Warmia and Mazury in Olsztyn, 10-719 Olsztyn, Poland; slawomir.kulesza@uwm.edu.pl

* Correspondence: boguslaw.pierozynski@uwm.edu.pl or bogpierozynski@yahoo.ca

Abstract:

This study investigates the influence of prolonged electrolysis on the electrochemical performance and surface characteristics of NiFe-modified compressed graphite electrodes used in alkaline water electrolysis. The electrochemical experiment was conducted over a two-week period at a constant temperature of 60 °C. Electrodes were evaluated for changes in surface morphology and composi-tion using scanning electron microscopy (SEM), energy-dispersive X-ray spectroscopy (EDS), and X-ray diffraction (XRD). The results demonstrated stable electrochemical performance with minimal current variation. However, significant structural changes occurred, including the formation of new microstructures on the cathode and the emergence of $CHKO_3$ (potassium bicarbonate) com-pound on both electrodes. Crystallographic analysis revealed an increase in crystallite size and tensile lattice strain on the cathode, while the anode exhibited compressive lattice strains and a reduction in crystallite size. These findings suggest that the observed changes are driven by elec-trochemical annealing processes, contributing to material redistribution and surface modifications during prolonged electrolysis. This study provides insight into optimizing NiFe-based catalysts for enhanced durability and efficiency in water splitting technologies.

Table S1. Various lattice parameters of the electrode materials obtained by means of the Scherrer and Williamson-Hall methods, where: (hkl) – is Miller index, 2θ – is Braggs' angle, FWHM – is full width at half maximum of the peak, D_S – is size of the crystallites evaluated using Scherrer method, D_{WH} – is size of the crystallites using Williamson-Hall method, ε – is relative lattice strains.

Sample	Crystalline phase	hkl	2θ [deg.]	FWHM [deg.]	D_S [Å]	D_{WH} [Å]	ε [10^{-2} %]	Vol. [%]
Graphite	Graphite (P63mc) a_0=2.4671 c_0=6.7579	002	26.355	0.2724	299.769	310.529	7.793	95.90
		100	42.265	0.3512	242.709			
		101	44.434	0.3601	238.497			
		102	50.498	0.3833	229.337			
		004	54.250	0.3665	225.302			
		103	59.590	0.4140	221.299			
		110	77.284	0.4606	221.001			
		112	83.328	0.4722	225.385			
	Graphite (R-3m) a_0=2.4601 c_0=33.4118	009	23.951	0.3121	272.871	290.011	6.841	4.10
		00.12	33.121	0.3316	249.505			
		012	42.752	0.3592	237.696			
		104	43.818	0.3622	236.599			
		110	77.546	0.4768	213.884			
		119	82.556	0.4989	212.057			
Graphite/NiFe	Graphite (P63mc) a_0=2.4677 c_0=6.7621	002	26.339	0.3738	218.444	219.954	9.635	100
		100	42.255	0.4844	175.963			
		101	44.422	0.4966	172.934			
		102	50.480	0.5280	166.475			
		004	54.215	0.5455	163.736			
		103	59.564	0.5682	161.221			
		110	77.263	0.6242	163.054			
		112	83.300	0.6353	167.486			
Cathode	Graphite (P63mc) a_0=2.4800 c_0=6.7465	002	26.400	0.2858	285.740	850.738	56.39	98.63
		100	42.036	0.6363	133.858			
		101	44.221	0.6712	127.857			
		102	50.325	0.7612	115.400			
		004	54.350	0.8159	109.538			
		103	59.464	0.8815	103.869			
		112	82.883	1.1489	92.315			
	$CHKO_3$ (P21/c) a_0=15.1736 b_0=5.6286	400	24.23	0.1759	-	-	-	1.37
		-111	28.781	0.1814				
		410	29.020	0.1816				
		-311	31.223	0.1841				

	c_0=3.7133	020	31.770	0.1847				
	β_0=104.6331°	120	32.364	0.1853				
		220	34.091	0.1871				
		211	34.325	0.1873				
		600	36.698	0.1897				
		-511	37.833	0.1908				
		401	39.240	0.1921				
		610	40.152	0.1929				
		411	42.520	0.1950				
		221	44.482	0.1966				
		720	54.522	0.2038				
		-203	77.363	0.2108				
		432	83.332	0.2096				
		-12.02	83.555	0.2095				
		133	101.779	0.1889				
		002	26.504	0.3749	220.702			
	Graphite	100	42.306	0.3784	225.293			
	(P63mc)	101	44.497	0.3779	227.315	157.645	-20.02	98.84
	a_0=2.4648	004	54.577	0.3689	242.514			
	c_0=6.7206	110	77.369	0.2830	359.907			
		112	83.478	0.2242	475.674			
		200	12.043	0.0300				
		110	16.862	0.1438				
**Anode**		400	24.221	0.2249				
	$CHKO_3$	-111	28.814	0.2595				
	(P21/c)	410	29.017	0.2608				
	a_0=15.1807	-311	31.242	0.2749				
	b_0=5.6261	111	31.418	0.2760	-	-	-	1.16
	c_0=3.7080	020	31.785	0.2781				
	β_0=104.6636°	-511	37.837	0.3095				
		401	39.274	0.3159				
		-221	40.658	0.3216				
		620	49.310	0.3502				
		-11.21	77.437	0.3509				
